# Art, Design and Communication Theory in Creating the Communicative Social Robot ‘Haru’

**DOI:** 10.3389/frobt.2021.577107

**Published:** 2021-03-01

**Authors:** Eleanor Sandry, Randy Gomez, Keisuke Nakamura

**Affiliations:** ^1^ School of Media, Creative Arts and Social Inquiry, Curtin University, Perth, WA, Australia; ^2^ Honda Research Institute Japan, Wako, Japan

**Keywords:** human–robot interaction, human–robot communication, art, design, design thinking, communication theory, human-machine communication

## Abstract

Haru is a social, affective robot designed to support a wide range of research into human–robot communication. This article analyses the design process for Haru beta, identifying how both visual and performing arts were an essential part of that process, contributing to ideas of Haru’s communication as a science and as an art. Initially, the article examines how a modified form of Design Thinking shaped the work of the interdisciplinary development team—including animators, performers and sketch artists working alongside roboticists—to frame Haru’s interaction style in line with sociopsychological and cybernetic–semiotic communication theory. From these perspectives on communication, the focus is on creating a robot that is persuasive and able to transmit precise information clearly. The article moves on to highlight two alternative perspectives on communication, based on phenomenological and sociocultural theories, from which such a robot can be further developed as a more flexible and dynamic communicative agent. The various theoretical perspectives introduced are brought together by considering communication across three elements: encounter, story and dance. Finally, the article explores the potential of Haru as a research platform for human–robot communication across various scenarios designed to investigate how to support long-term interactions between humans and robots in different contexts. In particular, it gives an overview of plans for humanities-based, qualitative research with Haru.

## Introduction

**FIGURE 1:**
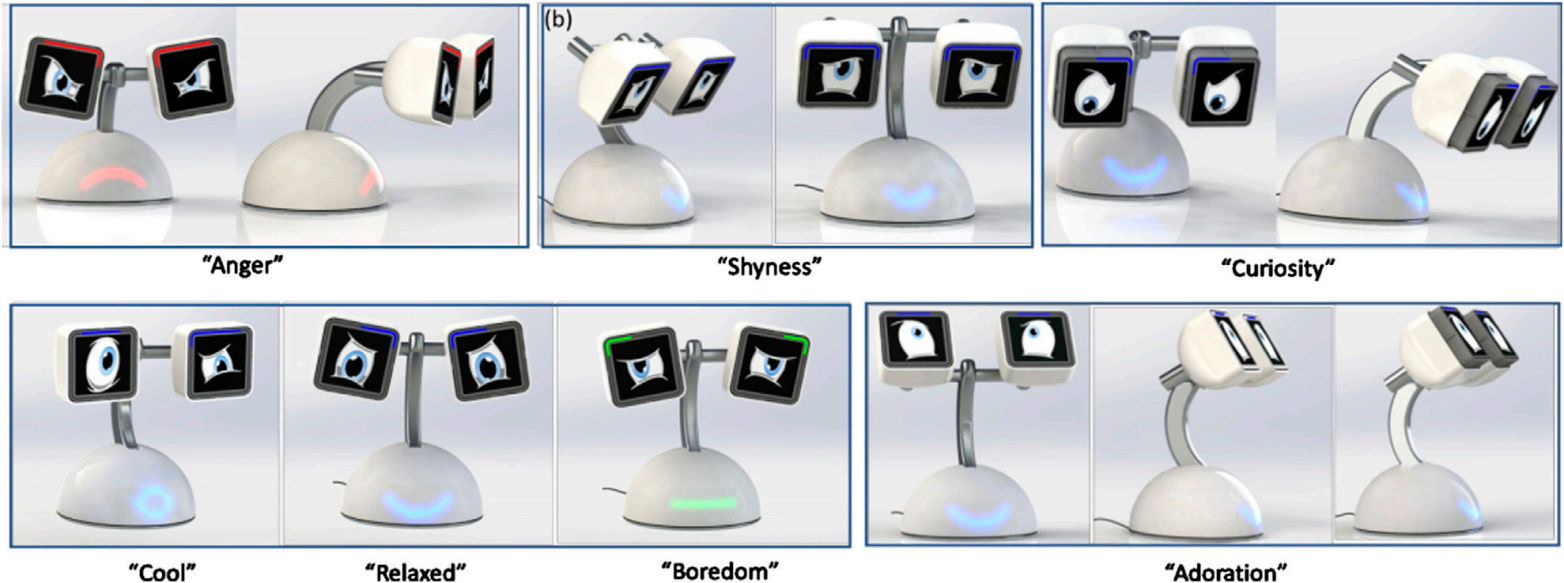
Haru beta showing some of its emotive affordances.

“Haru” is an experimental robotic platform developed to support research into human–robot communication from a number of disciplinary and methodological perspectives. The design for Haru’s first prototype, Haru Beta (Figure 1), concentrated on developing the robot’s physical form to include enough motion capability and other nonverbal affordances to support its emotional expression and communication in interactions with people (Gomez et al., 2018). The continued development of Haru will increase the robot’s capacity to communicate in a number of other ways that complement its nonverbal expressiveness, including using a voice and potentially via other novel affordances, such as content projected onto nearby surfaces. Haru is thus being developed with a broad idea of communication in mind, encompassing language, paralanguage and kinesics (Sandry, 2015). Haru’s flexible communication style is expected to support research into long-term human–robot interactions, since this robot’s multimodal communication has the potential to draw people into communication and sustain their interest over time. This article focuses on analyzing and theorizing Haru’s communication with people. Across different experimental contexts, Haru will likely be developed to use voice, facial and gesture recognition, allowing people to communicate with this robot using speech, facial expressions and bodily gestures.

The article begins by analyzing the design process for Haru Beta. It highlights how the work of visual and performing artists was integral in the development of this robot, while also examining how this shaped Haru’s non-verbal communication style in relation to sociopsychological and cybernetic-semiotic theory. It then moves on to consider sociocultural and phenomenological theory, which offer alternative perspectives from which this robot’s communication can not only be understood, but also developed further. The article therefore draws on a number of communication-theoretical traditions to examine Haru’s ability to interact with people, combining analyses of communication as a science and as an art. To organize this wide-ranging theoretical and analytical trajectory, the article frames its discussion of human–robot communication across three elements: first, people’s initial *encounter* with the robot, and how the robot might be recognized as a communicative other in this and subsequent meetings; second, ideas of *story*, drawing together the narratives that emerge not only as people interact with Haru, but also those told before, to frame the interactions, and after, to explain them; and third, *dance*, attending to the embodied nature of communication with Haru with the potential to support dynamic, overlapping verbal and nonverbal interchanges through which meaning emerges in interaction.

Having considered Haru’s communication in this way, the article introduces various communication scenarios that will shape future experiments, analyzing interactions between people and Haru in particular contexts. Experiments with Haru will use qualitative methods to complement and extend quantitative approaches more commonly used in human–robot interaction research. Alongside this, the article emphasizes how looking at the whole process of multimodal communication with Haru, across the elements of encounter, story and dance, sheds light on ways to create robots that not only attract people’s attention in the short term, but also are able to sustain meaningful communication in the long term, without becoming either irritating or boring.

## Analyzing the Design Process for Haru Beta

**FIGURE 2:**
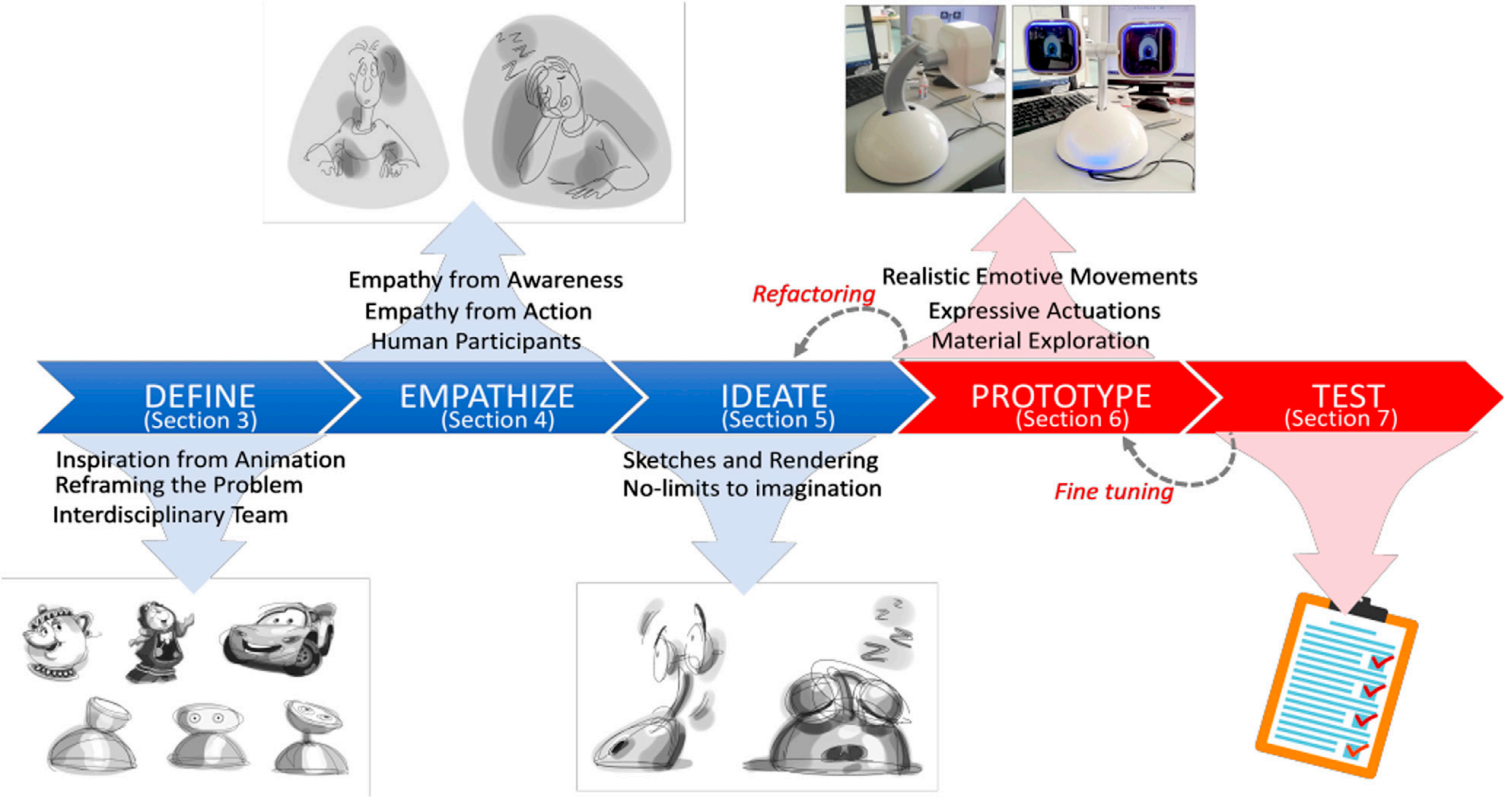
Design Thinking process adopted for the development of Haru Beta.

The design and development of Haru Beta used a customized Design Thinking model to outline a process able to accommodate an interdisciplinary team of animators, performers and sketch artists working alongside roboticists. Commonly, Design Thinking processes begin with an Empathize stage, during which designers take time to empathize and potentially also engage with prospective users of the product being designed, before moving into a Define stage that identifies a clear statement of the problem the design needs to address (Damand and Siang, 2019). In the case of Haru, a robot destined to be a platform for human–robot communication research flexible enough to work across a number of scenarios, disciplinary perspectives and methodologies, these two stages were swapped (Figure 2). This allowed the team to take the initial step of defining their own overarching problem driving this robot’s design; the need to build a distinctive, expressive and communicative robot that would support a high level of anthropomorphism without raising people’s expectations too high (Gomez et al., 2018).

Even in its modified form, as for the Design Thinking model more commonly used, the process for Haru’s development followed “an analytic and creative human-centered process” that cycled through stages involving “reflective thinking, productive action, responsible follow through and re-framing of the design problem” (Gomez et al., 2018, 235). An analysis of the design process for Haru Beta, focusing in particular on the Define and Empathize stages, follows. This identifies the perspectives on communication the process has a tendency to privilege. As will be explained, the human-centric nature of such a process has a tendency to reinforce the decision to make this robot communicate in ways that can easily be interpreted as familiar caricatures of human bodily and facial expressions (as well as potentially relying on readings of robots in relation to people’s prior experience of popular cultural texts, their pets and other animals). Later in the article, the benefits of complicating and extending this decision, in particular when a robot is expected to take part in interactions that are engaging for people over the long term, are considered.

### Define: Identifying the Initial Problem for Haru as a Communicator

As mentioned above, the project team pre-defined the initial problem they needed to address; how to create “an emotive, anthropomorphic tabletop robot” capable of sustaining “long-term human interaction” bearing in mind the likely build constraints for this machine, affecting the final look and feel of the robot, as well as its motion affordances (Gomez et al., 2018, 235). It is notable that the idea the robot should be “anthropomorphic” was stated up front, although the team was nonetheless concerned to retain an open mind as to the possibilities for the design, without becoming too bogged down in the likely physical issues of realizing this. Following the identification of the high-level goal, the Define stage continued by asking animators to produce sketches of various ways that Haru’s design might achieve this goal. This stage therefore drew on the skills of animators in making inanimate objects come “alive”. As the sketches in Gomez et al. (2018) show, animated characters from a number of popular films demonstrate how giving objects faces and making them bend and twist in ways impossible for those objects in the physical world creates animated characterizations that can be emotionally expressive in very humanlike ways.

The overarching assumption of this design path is that the expression of emotions via a recognizable face, most often with two eyes and other features that can be identified as eyebrows and a mouth, will help support easily read affective, and as a result engaging and effective, communication between human and robot. While Haru’s design team sought to “step away from a literal humanoid or animal form” (Gomez et al., 2018, 235), the anthropomorphic shaping of the resulting design is nonetheless very clear. As Figure 1 shows, Haru beta’s design includes two expressive eyes, animated on thin film transistor displays, with separate light emitting diode strips above that act as colored eyebrows. The eyes can be tilted, and each one can rotate and move in and out in relation to its casing. Finally, a light emitting diode matrix in the robot’s body is used to display a colored mouth of various shapes.

As well as supporting an anthropomorphic design path, the process of considering particular animation styles and techniques for normally inanimate objects in films and cartoons also shapes this robot’s communication in strongly sociopsychological terms. The sociopsychological tradition of communication theory regards communication as a form of information transfer, where the aim of the sender of a message is to persuade the receiver of something (Craig, 1999). In terms of robot design, this can be linked with the development of robots that can express emotions and are therefore likely to draw people into interactions often by being “cute” as seen with Kismet (Turkle et al., 2004) and Jibo (Caudwell and Lacey, 2019). Along similar lines, it is easy to see how Haru could also convey a cute personality.

The use of a cute aesthetic has been shown to work well to attract people’s attention toward interacting with a robot in the short term (Breazeal, 2002), but questions have been raised over how well this might work in the long term (Menzel and D’Aluiso, 2000; Caudwell and Lacey, 2019), the goal of the Haru project. In addition, although Haru has been categorized in some reports as designed for entertainment, where a cute personality might be particularly engaging and non-threatening (Zachiotis et al., 2018), this would likely not be appropriate when Haru is positioned to complete practical or business oriented communicative tasks. As discussed in more detail below, framing Haru’s appearance, expression and resulting personality using the Japanese term “kawaii” might offer a wider range of ways to consider this robot, not simply as cute, but also as playful, inquisitive and surprising, personality traits that might lend themselves to a robot positioned not just for entertainment, as well as suggesting how Haru might engage people in the long term with a personality that develops and changes over time.

Having come up with an overall concept for Haru, supported by a high-level goal and set of sketches showing Haru as an animated character, the team moved on to consider how Haru’s nonverbal communication would work in more detail. In particular, they were concerned to explore how Haru might express a variety of emotions at different levels of intensity and in ways that a wide range of people would recognize.

### Empathize: Expressing Emotion for/as Haru in Communication

In the Empathize stage of the design process, Haru’s design team worked with a set of volunteers, designated as performers. Having been shown the initial set of sketches for Haru from the Define stage, these people were asked to use a combination of body language and facial expression to act out particular emotions as they would themselves, and also as they imagined Haru would (Gomez et al., 2018). Although the Empathize phase of a Design Thinking process often involves empathizing with users, it is interesting to note that here the idea of asking users to try to empathize with the robot was also important, since this outcome will be a key part of Haru’s success. Video feedback was provided to the performers, and coaches gave instructions to help them express emotions across a range of different intensities (Gomez et al., 2018). Again, the strongly human centered nature of this process, with a focus on coding human expressions of emotion as well as asking humans to think themselves into the robot’s body imaginatively and mimic how the robot might emote, continues to support the anthropomorphic nature of Haru’s design. From a sociopsychological perspective, this robot is expected to attract attention and then persuade people to take part in continued interaction through its emotionally expressive communication and personality.

This process, involving the performance of emotional expressions, can be linked with François Delsarte’s method for acting, which is based on a close “analysis of facial and bodily gesture” to identify specific movements that can be reproduced to operate as an expressive language easily interpretable by an audience (Maltby, 2003, 400). In terms of communication theory, such an approach not only works in relation to the persuasive, sociopsychological perspective discussed above, but also supports how this style of emotional expression can be part of cybernetic-semiotic exchanges coded in intersubjective (catering in this case for human and robot understanding) language or other signs (theoretical structure from Craig, 1999, developed in Sandry, 2015). From a cybernetic-semiotic perspective the precise nature of the message and its clear encoding is key. Even without considering the use of verbal language, Delsarte’s approach to acting relies on a performer’s ability to code emotions into readily recognizable nonverbal facial and bodily expressions that precisely communicate specific emotional responses. For Haru’s design team, identifying ways to code emotion across a number of human bodies and faces assists in programming the robot to read people’s emotional state during an interaction. Then, asking performers to empathize with Haru, putting themselves in the robot’s place to act out how they think it would express an emotion, supports the design of the robot’s expressive eye animations and movements of its eyes, eyebrows, neck and mouth.

### Realizing Haru Beta in Physical Form Through Ideate, Prototype and Test Phases

The human-centered Define and Empathize phases of Haru’s design process drove a strongly anthropomorphic conceptualization for Haru. The importance of supporting anthropomorphism continued into the Ideate phase of project, with its focus on finding imaginative solutions to make Haru as expressive as possible, setting aside likely practical limitations for a physical robot. At this point sketch artists built upon the early sketches for Haru created in the Define phase to show the potential for the robot to express emotions inspired by the findings of the Empathize process. These sketches reinforced the anthropomorphic shaping of Haru throughout the design process, and also clearly raised some challenges for the physical prototype.

While a sketch artist can stretch and squash a robot’s body in extreme ways that reinforce particular emotional expressions, this is difficult to realize for a physical robot that already has a relatively complex design. The Prototype phase therefore necessarily involved an iterative process, through which the ideas developed during ideation were taken seriously, but also refactored in light of the limitations of the physical and engineerable form the robot would take. Following the development of a prototype, a Test phase allowed the performance participants to offer feedback on the design enabling further refinement. As Gomez et al. (2018) note, this phase was not meant to encompass a full user interaction study, but rather was an integral part of the initial design process to test the effectiveness of Haru’s emotional expressions.

### Design, Art and Communication as a Science

The development of Haru Beta made good use of a modified Design Thinking process to shape and coordinate the work of an interdisciplinary team, including those with skills in visual and performing arts. In particular, the artists’ conceptions for the expressive possibilities of this robot, as well as the ability of performers to produce their own expressions, and expressions “as Haru”, provided a depth and complexity to conceptions of this robot as an expressive communicator. These then had to be tempered by roboticists as engineers well aware of the physical constraints of building a robot.

Although design and art were key to Haru’s development, the analysis above shows how the robot’s resulting communication can nonetheless be framed in scientific terms, as precise, clearly coded and reliably persuasive for anyone with whom it interacts. It would seem to make a great deal of practical sense to design and develop with sociopsychological and cybernetic-semiotic communication success in mind. Such a focus is easily judged likely to create a robot that is compellingly cute, familiar and easy for humans to interact and communicate with.

## Extending a Broader Conception of Haru’s Potential as a Communicator

However, thinking about human-human communication, let alone human–robot communication, in anything other than the simplest situations highlights the difficulty of perfectly coding precise messages, or of reliably persuading the other, because the majority of communicative events involve some level of ambiguity, together with the potential for misunderstanding. Indeed, scholars have argued that ambiguity and misunderstanding are actually an intrinsic part of any worthwhile human communication (Bennington, 1994; Chang, 1996). While ideas of communication as a scientific and perfectible process might be attractive, it is therefore also useful to embrace the art involved in communication that recognizes the value of a more relational and dynamic understanding of communicative processes within which one must also acknowledge the impossibility of comprehending the other completely.

This idea is emphasized by the phenomenological tradition of communication theory for which any attempt to *know* or understand the other fully is fraught with difficulty. Instead, a phenomenological perspective emphasizes the other’s difference from the self as a chasm that cannot be bridged by an empathetic stance (Levinas, 1969; Craig, 1999; Pinchevski, 2005). This perspective raises questions not only in relation to understanding the communication of a robot such as Haru, but also for the design process discussed above, which asks performers to empathize with Haru and act out how this robot might express a particular emotion. It is therefore good to note that when working with the results of the Empathize stage, Haru’s creators embraced the way this robot’s eyes and neck had the potential also to express with movements similar to a person’s hands, arms and shoulders (Gomez et al., 2018). This opens up broader possibilities for Haru’s expression to be both *like* that of a human, and also *fundamentally different* (given its very different form and potential to express in non-humanlike ways that are nonetheless read by humans as communicative).

The more philosophical take on communication offered by phenomenological theory conveys an idea of otherness that is not only open to the difficulties of making a robot with non-humanlike form express in humanlike ways, but also one that suggests a robot other’s difference is an integral part of why one might communicate with it as opposed to a problem that must be overcome (Sandry, 2015). Framing the initial moment of meeting as an encounter with otherness draws out the importance of difference as a means not only of attracting attention, but also retaining attention over the mid to long term. As Caudwell and Lacey suggest (2019, 10), it may well be important for social robots to “maintain a sense of alterity or otherness, creating the impression that there is more going on than what the user may know”, because “this sense of alterity (real or simulated)” is one way to break down “the strict power differential that is initially established by their cute aesthetic”. In this way, a social robot can become more than a compliant communicator always focused on responding to human queries and questions; instead, such a robot can be recognized as having the potential to act on its own, provide information or call for human attention and response as it requires.

### Framing Haru as “Kawaii”

Extending these ideas further, rather than framing Haru’s personality and expressive ability as “cute”, as mentioned above, it may be more productive to adopt the Japanese term, “kawaii”. Although this term is often translated as, or at least closely associated with, the English word “cute” and its meaning (Cheok and Fernando, 2012), describing Haru as kawaii draws attention to this robot’s potential for playful communication, approaching things with what seems to be “an inquisitive attitude” and the ability to surprise users in interactions, catching them “off guard” (Cheok and Fernando, 2012, 300). The idea of Haru’s ability to surprise people resonates with the importance of “interruption” in phenomenological perspectives on encounters between selves and others (Pinchevski, 2005; Sandry, 2015). Even as self and other are drawn into the proximity of an encounter, “the face to face”, the other’s alterity is always a factor (Levinas, 1969). From this perspective, Haru retains the potential to interrupt or surprise people by expressing itself as a social, communicative, other-than-human presence. As opposed to being non-threateningly familiar, Haru thus has the potential to be quirky and unusual, drawing people’s attention and inviting their participation in continued communication. Alongside the potential to be surprising though, Haru’s small size relative to humans (even a human child) and the robot’s fixed positioning nonetheless mean this robot is unlikely to scare anyone away.

It should be noted that defining Haru as kawaii, or even as having kawaii characteristics, is complicated by the fact that different people, and even the same person across changing circumstances, may or may not choose to appraise the same object as kawaii (Nittono, 2016). Even while a kawaii robot might be a more intriguing communicative partner than one regarded simply as cute, there is likely still a delicate balance between it being delightfully quirky, or irritatingly inappropriate. The shifting attribution of the term kawaii depending on the preference of individual people and changes in context for encounters with Haru raises the importance of considering a sociocultural perspective on communication in human interactions with this robot. This perspective analyses communication as a means of producing, reproducing, and negotiating shared understandings of the world (Carey, 1992; Craig, 1999). From this perspective, communication is heavily reliant on the overarching cultural setting as well as the detailed context of an interaction between particular individuals. The space within which people interact with Haru, the framing of the robot’s purpose, how familiar a person is with the robot, the presence of other people that can see and hear the interaction as bystanders, watching someone else interact with the robot, previous experiences with interactive technologies and many other factors may well have an appreciable effect on how people will respond to the robot (a number of these factors being raised in Lee et al., 2010). Some of these contextual elements can be thought of in terms of narratives, including stories told to situate Haru in relation to a particular communicative scenario, the stories that may emerge in interaction and also the stories that people see played out in other’s interactions with the robot, or those they hear other people recount about their experiences with the robot. The importance of context, and the idea of changing appraisals of a robot, also highlights how human–robot interactions are dynamic, not just within the interaction itself, but also in relation to the situational factors that surround that interaction. It is not only the story that emerges within an interaction that is important, but also the surrounding stories that shape and frame the interaction in particular ways.

### Communication as a Dynamic Process Occurring in a Dynamic System

A consideration of sociopsychological, cybernetic-semiotic, phenomenological and sociocultural perspectives on interactions with Haru suggests that it is useful to adopt a more dynamic understanding of communication overall, which could be important across design and prototyping contexts, as well as in planning user interaction studies with robots. In particular, although Delstarte’s idea of coding emotions for performance is a practical part of the design process discussed in this article, considering how emotional communication can emerge through dynamic interchanges highlights the potential for an alternative acting paradigm to play a part. This alternative view is typified by the Stanislavski technique, within which performers are expected to coordinate with one another in the moment of interaction, behaving in ways shaped as reactions or responses to other performers (Moore, 1960; Hoffman, 2007). When human–robot interactions are considered from this perspective, the precise coding of emotion or of information becomes less important than the ability of the robot to respond to changes in its environment as well as to the particular person with which it is currently engaged in interaction.

From a dynamic systems perspective, communication is not about the transmission or exchange of fixed pieces of information, because, as Alan Fogel argues, “information is created in the process of communication”, such that “meaning making is the outcome of a finite process of engagement” (2006, 14). This shifts a cybernetic-semiotic focus from a preoccupation with clear and precise messages, to considering the value of iterative exchanges of feedback and response through which meaning emerges (Sandry, 2015). It also reinforces the idea that sociopsychological persuasion may rely not so much on any fixed perception of “cuteness”, but rather on reading a personality that develops and changes within and between interactions. Finally, a focus on dynamic communication, or communication as a form of “dance” (Shanker and King, 2002, 605), draws attention to the potential for nonverbal, embodied communication to support exchanges that are not restricted by turn-taking, but rather become continuous processes “within which signs can overlap even as they are produced by the participants” (Sandry, 2015, 69).

Although it would have been difficult to assess the dynamics of particular interactions with Haru in the early stages of the design process discussed above, this approach should become easier to plan for and to apply once a working prototype becomes more widely available to allow people to test what it is like to interact with Haru directly. In addition, while the development of Haru Beta has concentrated on designing the robot’s body to allow expressive emotional communication, this robot will also need to communicate flexibly in a range of other ways if it is to fulfill the goal of being a platform to support human–robot communication research more fully.

### Developing Haru’s Communication across “a Triple Audiovisual Reality”

The conception of Haru’s potential as a flexible, dynamic communicator developed above identifies the need to incorporate more communicative skills for this robot than the nonverbal expressions developed for Haru Beta. Overall, the development of Haru, as a social robot for long term interaction, is likely best driven by a broad understanding of what constitutes communication as “a triple audiovisual reality” (Poyatos, 1997). This idea is drawn from research into the complexity of simultaneous translation, which emphasizes the need to attend to not only the words people use, but also a range of communicative elements that surround those words, in order to come close to an accurate translation of what someone is saying. The triple structure, when concerned wholly with human communication, consists of verbal language (speech itself), paralanguage (tone of voice, nonverbal voice modifiers, and sounds), and kinesics (eye, face, and body movements) (Poyatos, 1983).

From this perspective, Haru Beta’s communication design focuses entirely upon kinesics through its eye animations and movements, eyebrow colors and shapes, neck movements, and colored light displays on its body. It should be noted that in some cases the kinesic communication of the robot amounts to a direct communication signal, such as a red down-turned mouth on its body, combined with frowning eyes and red eyebrows, which can be read as a clear coding for anger (drawing on Delsarte’s ideas on communicating emotion through acting). In contrast, some of Haru’s other kinesic expressions may be less obviously coded, more ambiguous and open to interpretation on the basis of context. This applies in particular to Haru’s more complex emotional expressions shown in Figure 1, such as shyness and curiosity. Currently, the animation of Haru’s eyes offers the most flexible mode of expression, but attempts to make the robot’s other features more subtly expressive may be made in future.

Haru’s continued development has involved the introduction of a voice interface, although the exact voice Haru uses could be refined on the basis of user-interaction studies. With its vocal capabilities, Haru can communicate across the triple structure in face-to-face situations in language and using expressive sounds, as well as through its body movements. Haru’s other-than-human form also has the potential to support completely novel modes of communication, such as expressing emotion through colored lights, the ability to project content onto a wall or screen, and maybe other forms of body language (dependent on the final form of the robot).

### Considering the Art of Communication in Relation to Robots such as Haru

In spite of the fact that creative art and design played a key role in the development of Haru beta, the goal of creating an easily anthropomorphized, communicative robot was linked earlier in this article with scientific ideas about precisely coded emotional expressions that support both sociopsychological and cybernetic-semiotic understandings of this robot’s communication. More recent sections of the article argue that more complex communication scenarios likely involve ambiguity, the potential for misunderstanding, and the need to adopt a more dynamic understanding of communication during which meaning emerges over the course of an interaction. This idea might be particularly important for robots designed to communicate with people over a sustained period or on a number of separate occasions.

The question of how a robot might encourage people to interact with it repeatedly or in the long term is not simple to answer. Guy Hoffman (2019), for example, when considering robots designed to share people’s homes identifies “clear technological barriers” in relation to both “realistic non-repetitive gesture generation” and “dialogue algorithms” that make supporting sophisticated interactions over time difficult. This is clearly an important consideration for social robots more generally (whether in homes, workplaces or social spaces). Rather than assuming that robots need to be more complex or intelligent, Hoffman argues that social robot development needs artists, “professionals who excel at storytelling, emotional engagement, and structured repetition” (2019). In particular, Hoffman goes on to identify the value of taking seriously the development of stories within and around interactions between humans and robots.

These ideas are being put to the test as Haru’s development continues, with the team actively exploring how the development and performance of “captivating storylines” might help a robot to attract people’s attention and encourage engagement over the long-term. The idea of content creation for Haru in part relates to developing stories Haru might tell people, potentially projecting graphics onto nearby surfaces. While Haru might well tell stories designed to entertain, alongside this the idea is for Haru to share its own story, giving people a sense of Haru’s internal life and imagination, potentially adding creative depth to people’s sense of Haru’s existence and personality. The content Haru projects onto surfaces, for example, will therefore be designed not to reduce people’s attention to Haru, but rather to complement Haru’s presence, giving the robot a context from which to be understood, a meaningful back-story. The importance of back-story can be seen in the development of other robots, such as Fish and Bird based on characters from a Greek myth (Velonaki et al., 2008), for which a story not only supports the development of the individual characters of the robots, but also helps to explain their interaction with each other for visitors to their installation space. In the case of Haru, the idea of content creation in the form of storytelling is expanded. This robot is able to tell its own story, as well as stories designed to entertain, but, maybe more importantly, people’s interactions with Haru are also thought of as creating their own stories that develop over time.

The move from understanding communication as a process of coding signals, whether in language or through nonverbal sounds and signs, to viewing communication as a dynamic and constantly emerging interchange between communicators (whether human and human, or human and robot), can be framed as a move away from scientific ideas about communication as a perfectible process and toward acknowledging the art of communication. Historically, it is the rhetorical tradition that regards communication as the “practical art of discourse” (Craig, 1999, 135), but it has been argued that “there is considerable overlap between the rhetorical tradition and others” allowing this idea to be extended to communication as defined more broadly (Littlejohn and Foss, 2011, 62). From this perspective communication is less about a fixed message and more about a developing story, where conversations can link back to previously shared experiences, such that memory and the continual emergence of new meaning combine to support interactions perceived as valuable over the long term.

## Future Scenarios for Research with Haru to Explore Communication in Context

At this point, two overarching scenarios are being developed and implemented to drive future phases of research with Haru. The first of these positions Haru as a robot that supports a new form of hybrid telepresence. In this scenario, the initial emphasis will be on using Haru as a novel interface, to add a level of expressiveness when someone at a distance is communicating through the robot using either text or voice. Clearly this is most important when the person communicating cannot provide a video feed; however, even when a video feed is supported, it can be argued that the addition of a means to support gestural and body language could enrich telepresence, in particular when the telepresence user is trying to communicate with a group of people (Stahl et al., 2018). Development of teleoperation interfaces for Haru serve a double purpose, allowing research into how this type of affective robotic platform can extend a person’s telepresence without or alongside a video display, but also providing the opportunity to test the range of Haru’s expressiveness prior to developing its capability for autonomous operation, the second scenario for research with Haru. In this scenario, Haru becomes a communicator in its own right. Whether Haru is positioned in a home, workplace or other social context, the aim is that the robot will communicate in a way that is immediately engaging and conveys a sense that it has a clear personality. As this article has discussed, the idea is that people want to communicate with Haru not only on first meeting the robot, but also on subsequent occasions, such that they are drawn into long-term interaction. At times, Haru’s ability to communicate pertinent information clearly will be vital, but the development also embraces a broader idea of communication that encourages people to respond to the robot as an entity with which they are happy to engage on many occasions.

### Haru as a Negotiator and Mediator Assisting People in Telepresence Communications

When positioned for telepresence, unless two Haru robots are in use, one person’s encounter with Haru will be mediated through a smart device or computer interface, whereas the other person will interact directly with the robot. This scenario therefore involves development and testing of a digital interface for Haru, where the suggestion is that text and speech will be augmented through the use of “Harumoji”, emoji that convey Haru’s particular embodiment and expressive style. Harumoji might well be used to help the person at a distance control Haru’s physical expression, to add depth to the expressive quality of the communication possible through the telepresence interaction with the person in front of the robot. In considering this scenario, there is a sense that the person with Haru will already be familiar with the robot, its form and embodied communications. This highlights an important question for experiments with Haru for this scenario: how will Haru negotiate the move from *communicating with a person as itself*, for example to gain their attention and let them know someone wants to communicate with them, to *communicating as the person calling from a distance*? While the narrative frame to initiate people’s understanding of Haru as a telepresence assistant might seem simple, the narrative within the interaction itself will need to negotiate gracefully the changeover from Haru expressing its own agency and communicating for itself in interaction, to Haru providing a telepresence service, expressing and communicating on behalf of a person, and back again once the telepresence call is complete.

It is worth noting that for this scenario, the novel communication channels Haru might support, such as projection onto nearby surfaces, could either be used to support video of the person communicating at a distance, or may prove more useful when displaying materials being discussed in the conversation. The potential of the embodied aspects of Haru’s communication are really the key element of this scenario, designed to explore how Haru’s expressive physical form could be used to add depth to, or complicate, the communication of emotion in someone’s tone of voice through its expressive eyes, eyebrows, neck and mouth. Importantly, as mentioned earlier in this article, the design process for Haru also identified that this robot’s body, in particular the eyes and neck, can convey a sense of a person’s expression via hands, arms and shoulders. In this scenario, a level of human control over the robot’s expressive communication would be maintained much of the time, so it offers the opportunity to explore Haru’s potential as a communicator through a wizard-of-oz process that is not hidden from human participants, but rather is overtly presented as part of the experiment.

### Haru as a Communicator with People in its Own Right

Working to extend Haru’s control of its own communication (in part developed through working with Haru for telepresence) subsequent scenarios will be concerned with building Haru’s ability to develop and express a personality, whether Haru is positioned as a receptionist for a business, information provider in public spaces, or operating in the home as a personal assistant or companion. In smart homes and workplaces, Haru might also be integrated to assist with managing systems and devices in the surrounding environment. The eventual aim will be to enable development and expression of a personality associated with Haru’s robot agency, which will make this robot seem somewhat “alive” to people during an interaction. Overall, the goal is to support Haru’s ability not only to communicate, but also to build relationships with people over time, as the robot interacts and collaborates with them in shared activities.

As mentioned earlier in this article, while Haru might be relatively successful as a “cute” personal assistant in the home, this type of robot has not yet proved successful (the examples of Jibo and Kuri, also discussed by Hoffman (2019), spring to mind). It may be that Haru would be better off with a personality that conveys more depth even during the initial encounter, and certainly one that develops beyond that framing over time. In business, workplace and public settings where Haru is positioned as providing a service, whether as a receptionist or information provider, it is likely that in most contexts people might prefer to meet Haru as friendly in professional, as opposed to personal, terms (Sutherland et al., 2019). This is something that a cute aesthetic might not support that well at all. In addition, Lee et al. (2010) note that different people are likely to encounter the robot in different ways shaped by their previous experience. While some will be open to taking part in a friendly conversational exchange, others might prefer to treat Haru as an information providing machine, with no need to engage in social niceties. This may pose a challenge for Haru’s communication style, although the clearly socially communicative nature conveyed through its form for anyone encountering this robot even for the first time, may go a long way to encourage friendly interactions on most occasions.

Whether Haru operates in a home, workplace or public space, it is likely that this will require communication not just in response to an initial encounter, but also over time with people who frequent the space the robot occupies on a regular basis. An exploration of how operation over the long term might best be supported is one of the core reasons Haru has been developed. Other studies of robots positioned in a space over the long term provide some insights. In particular, Simmons et al. (2011) identify the need for the robot to have a background story, and also some sense of a life story that can be revealed over time. This provides a context for the robot’s actions and also supports how they might change over time, this change being important in sustaining people’s interest and engagement with the robot in the long term.

As discussed in a previous section, Haru’s project team has noted that the potential for Haru to project images onto surfaces might become a part of an embodied communication of Haru’s background story that could also provide a sense of life story over time. In addition to Haru being positioned in physical spaces with people, this robot could also project and interact with its own virtual world. Haru’s interactions with its virtual world would be used to reinforce the sense that Haru is somehow “alive”, even when it is not taking part in an ongoing interaction with a person, allowing people’s perceptions of its personality to develop along with the visual story the virtual space supports. From a wholly practical perspective, Haru’s interactions with this virtual space would also allow clear communication of when Haru’s attention is on its world (the projection being brighter and in focus) and when Haru turns its attention to a nearby person (the projection becoming faded and out of focus).

### Exploring the Shifting Sense of Agency in Communication with Haru

Haru’s communication with people across the scenarios described above, from telepresence to robotic agency taking part in shared activities across different contexts, can be understood from all the different communication-theoretical perspectives introduced in this article. Initially, meeting Haru can be framed as an encounter, which invokes the sense in which Haru expresses a personality, an otherness and also a level of apparent agency. At times, addressing Haru’s ability to communicate precisely and clearly in cybernetic-semiotic terms will be vital, in particular when Haru needs to provide someone with information directly. At other times, Haru’s communication might be quickly understood from a sociopsychological perspective, for example as the robot attempts to persuade people to interact with it in particular ways through its expressive communication, most likely trying to elicit a friendly and sociable tone. As Haru is embedded in particular contexts (such as being a receptionist or personal assistant), but even before this with Haru firmly positioned as a robot people meet through laboratory experiments, a sociocultural perspective on communication draws attention to the specific context and the details of how Haru is situated alongside the people with which it interacts. An understanding of Haru’s communication may well be shaped by people’s existing expectations of a social robot, but the real task may be to allow Haru to build on those expectations to enable new ways to communicate, developing people’s sense of its unique personality and ability to take part in shared activities with them. This leads to the importance of supporting Haru as seeming to be, at least somewhat, “alive”, a social being which people initially encounter and also continue to want to interact with, as a potentially surprising other from a phenomenological perspective, but also as an other that has its own life story conveyed through a novel, embodied communication channel involving projected content.

### Methodologies for Gauging the Success of Haru’s Communication in the Long Term

An exploration of Haru’s potential as a communicator, and the potential for human–robot communication more broadly, can clearly be driven from a number of experimental and analytical directions, including those that draw on techniques and methods from psychology, human–computer interaction (HCI) and human–robot interaction (HRI) studies. The Haru project team also recognize that these can be complemented by a humanities perspective, in particular when the goal is to assess how human–robot communication might develop over an extended period of time.

When considering the potential for research considering long term HRI, for example in a person’s house, it has been noted how “few studies have investigated the long-term use of technological systems in home environments”, meaning “the traditional technology acceptance literature lacks a profound body of long-term research” (de Graaf et al., 2018, 2583). It is clear though that such research would be valuable across any context where a human and robot were expected to interact in the long term, since it seems likely that “the development of user experiences with a technology or gaining user skills might change the user’s attitudes toward, uses of, or even the user’s conceptualizations of that technology” (de Graaf et al., 2018, 2538 citing a number of research studies in relation to each of these potential changes). One of the reasons for the lack of long-term HRI research studies may well be that “robot technologies are generally not robust enough to be studied outside the lab for extended periods of time without supervision of an expert” (de Graaf et al., 2017, 224). As a research platform, not a commercial robot designed for consumers, this is clearly an issue faced by researchers using Haru, but a consideration of long-term interaction is explicitly stated as one of the project’s goals. One way to carry out this type of research is to engage with humanities methodologies, such as autoethnography. As the “auto” prefix suggests, the advantage of pursuing this methodology in particular is that the robot remains in the care of the researcher.

While humanities methods tend not to offer quantitative measurements as results, their value is in the added depth and breadth of understanding they provide by developing theoretical and qualitative explanations of what happens in and around human–robot interactions. For example, adopting an autoethnographic framework for research with Haru will allow the researcher to write about their own experiences with the robot, developing detailed “thick descriptions” of what it is like to interact with and through Haru. This type of research can be conducted over a planned period of time that might span a few days, weeks or months. Although autoethnography is not often used as a methodology for HRI or social robotics research more generally (Chun, 2019), there have been some recent exceptions. For example, Verne (2020, 41), writing about adapting to using a robot lawn mower, explains how “autoethnography as the methodology gave rich access to events and personal experiences”, valuable because “personal thoughts and reflections were important for understanding how [they] changed [their] goals and values while adapting to the robot”. Clearly this idea resonates with the potential de Graaf et al. (2018) see in carrying out long-term research projects, while also mitigating the potential lack of robustness in the robotic platform. The use of thick description is also open to noticing and documenting all elements of the interaction with Haru, including the initial and subsequent encounters with the robot as an other, the stories that are told in and around interactions with and through the robot, as well as the details of the embodied dance of communication that any interaction with Haru will entail.

Although some researchers might argue that such research has limited use, since its “findings cannot be extrapolated to larger populations” (James et al., 2019, 2.8), it can certainly drive future research involving participants interacting with Haru, forming the basis for observational studies as well as semi-structured interview questions for participants (the latter being a use James et al. (2019) acknowledge). In addition, the published research of Verne (2020) discussed above, as well as positive reviews of larger projects such as *Seeing like a Rover*, where thick description is used to convey the responses of mission scientists to mars rovers (Vertesi, 2015), highlights the value of this type of qualitative observation and recording of people’s responses to robots in its own right.

## Conclusion

As this article has shown, adopting a Design Thinking methodology for an interdisciplinary team of animators, performers, sketch artists and roboticists, embraces ideas from both design and art. This design path results in the creation of a robot that is strongly framed as anthropomorphic, potentially also with a cute aesthetic. During the design process, there is also likely to be a focus on the robot’s sociopsychological and cybernetic-semiotic communication capabilities, theories of communication that can be associated with scientific ideas of communication as a perfectible process of precisely coded information exchange or toward successful persuasion. However, drawing on phenomenological and sociocultural theory, and employing the idea of a robot as kawaii as opposed to cute, provides a broader conception of the potential for Haru as a communicator open to a more relational and dynamic understanding of the art of communication, within which it is vital to respond to the other and their difference from the self. This is further reinforced by engaging with ideas of communication that encompass language, paralanguage and kinesics. This triple structure is important during initial and repeated encounters with Haru, as well as in relation to the sociocultural, ideological and narrative contexts, or stories, in and around that interaction. Acknowledging communication as more than language also highlights the importance of embodied and dynamic approaches that position communication as a dance of interaction.

As a platform for communications research, it is clearly important that Haru’s design not only lends itself to the broad analysis presented here, but also that the development team will use that analysis to drive future research and potentially also new design decisions. This article’s argument suggests that there are benefits to considering communication theory of many types in all robot developments to support the creation of machines that are flexibly able to communicate in many different ways, and that have the potential to be interesting communicative companions even in the long term. The article has also highlighted how humanities research methods, with a focus here on autoethnography, offer valuable qualitative techniques that can complement and extend the quantitative methods more often used in research that investigates human interactions with robots.

## Data Availability

The original contributions presented in the study are included in the article/Supplementary Material, further inquiries can be directed to the corresponding author.
